# Alongshan Virus Infection in *Rangifer tarandus* Reindeer, Northeastern China

**DOI:** 10.3201/eid3007.231219

**Published:** 2024-07

**Authors:** Wenbo Xu, Wei Wang, Liang Li, Nan Li, Ziyan Liu, Lihe Che, Guanyu Wang, Kaiyu Zhang, Xianmin Feng, Wen-Jing Wang, Quan Liu, Zedong Wang

**Affiliations:** Department of Infectious Diseases and Center of Infectious Diseases and Pathogen Biology, First Hospital of Jilin University, Changchun, China (W. Xu, Z. Liu, L. Che, K. Zhang, Q. Liu, Z. Wang);; Gansu Agricultural University, Lanzhou, China (W. Wang);; Hulunbuir Animal Disease Control Center, Hailar, China (W. Wang, G. Wang);; Changchun Veterinary Research Institute, Chinese Academy of Agricultural Sciences, Changchun (L. Li, N. Li, Q. Liu, Z. Wang);; Jilin Medical University, Jilin, China (X. Feng);; Beijing YouAn Hospital, Capital Medical University, Beijing, China (W.-J. Wang)

**Keywords:** viruses, vector-borne infections, Alongshan virus, reindeer, *Rangifer tarandus*, infection, China

## Abstract

We investigated Alongshan virus infection in reindeer in northeastern China. We found that 4.8% of the animals were viral RNA–positive, 33.3% tested positive for IgG, and 19.1% displayed neutralizing antibodies. These findings suggest reindeer could serve as sentinel animal species for the epidemiologic surveillance of Alongshan virus infection.

The novel tickborne virus Alongshan virus (ALSV) belongs to the Jingmenvirus group of the *Flaviviridae* family and is associated with human febrile illness (*1*). Initially identified in tick-bitten patients and *Ixodes persulcatus* ticks in the Greater Khingan Mountains of northeastern China (*1*), ALSV has since been identified in *I. persulcatus* and *I. ricinus* ticks in various locations, including Russia (*2*), Finland (*3*), Switzerland (*4*), and Germany (*5*). ALSV-specific antibodies have been detected in game animals, such as roe deer and red deer, as well as in domestic animals such as cattle, sheep, goats, and horses (*5*,*6*).

Semidomesticated reindeer, primarily raised by the Ewenki people in the northern Greater Khingan Mountains of China, are the predominant animals in this region (*7*). The reindeer serve as blood meals for ticks and could potentially act as reservoir hosts for tickborne pathogens. However, the extent of ALSV infection in reindeer remains largely unexplored. In this study, we investigated the RNA viromes of reindeer and their parasitic ticks, offering molecular and serologic evidence for ALSV infection in tick-exposed reindeer. The research protocol for this study was approved by the Animal Administration and Ethics Committee of the First Hospital of Jilin University (Changchun, China).

## The Study

**Figure 1:**
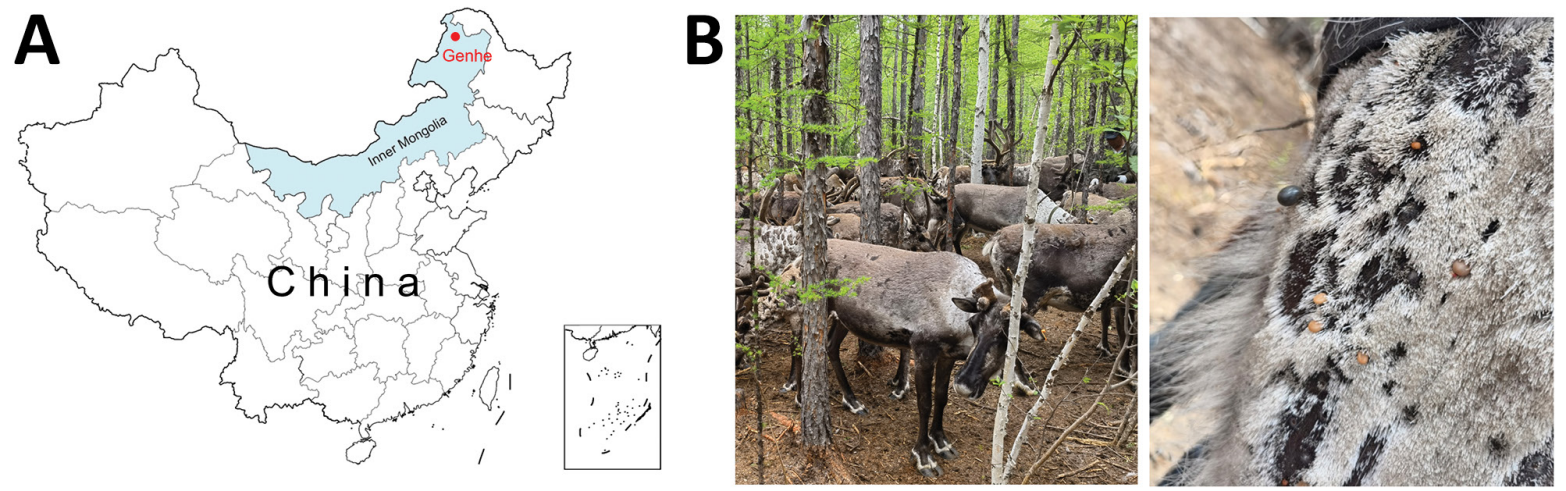
Sample collection process in study of Alongshan virus infection in *Rangifer tarandus* reindeer, northeastern China. A) Collection site of reindeer serum samples and their parasitic ticks. B) Sampled reindeer group and the presence of ticks on a reindeer.

In July 2022, we collected a total of 21 reindeer serum samples from Genhe in the Greater Khingan Mountains in northeastern China (Figure 1, panel A). In addition, we collected 93 bloodsucking ticks from the reindeer, morphologically identified them as *I. persulcatus* ticks, and grouped them into 13 pools on the basis of tick sex and size (Figure 1, panel B) (*8*). We pooled the reindeer serum samples and tick lysate supernatants separately, treated them with micrococcal nuclease (New England Biolabs, https://www.neb.com), and extracted viral RNA using the TIANamp Virus RNA kit (TIANGEN, https://en.tiangen.com). We sent the samples to Tanpu Biologic Technology in Shanghai, China, for metatranscriptomic analysis as previously described (*8*).

The sequencing process resulted in 5.2 GB of clean data and 38.5 million non-rRNA reads for the reindeer serum library, as well as 6.0 GB of clean data and 45.0 million non-rRNA reads for the tick library. From the reindeer serum library, we identified only 1 contig sequence related to ALSV. In contrast, the tick library revealed a total of 64 tickborne viral contigs. Those viral contigs were further annotated, revealing their association with 7 distinct viruses across 5 viral families. The identified viruses consisted of ALSV and tickborne encephalitis virus (TBEV) from the *Flaviviridae* family, Beiji nairovirus (BJNV) from the *Nairoviridae* family, Sara tick phlebovirus (STPV) and Onega tick phlebovirus (OTPV) from the *Phenuiviridae* family, and Nuomin virus (NUMV) from *Chuviridae*, and Jilin luteo-like virus 2 (JLLV2) from the *Solemoviridae* family (Table 1).

**Table 1:** Tickborne viruses identified in study of Alongshan virus infection in *Rangifer tarandus* reindeer, northeastern China

Classification	Virus species	Closest relative strain (% nt identity)
Flaviviridae		
Jingmenvirus	Alongshan virus	NE-TH4 (96.9–98.7)
* Orthoflavivirus*	Tick-borne encephalitis virus	HLB-T74 (96.6–98.4)
Nairoviridae		
* Norwavirus*	Beiji nairovirus	NE-SL3 (99.6)
Phenuiviridae		
* Ixovirus*	Sara tick phlebovirus	NE-SL3 (99.3)
	Onega tick phlebovirus	NE-SL3 (99.1)
Chuviridae		
* Mivirus*	Nuomin virus	SL4 (99.6)
Solemoviridae		
Sobemo-like	Jilin luteo–like virus 2	DH3 (98.5)

**Figure 2:**
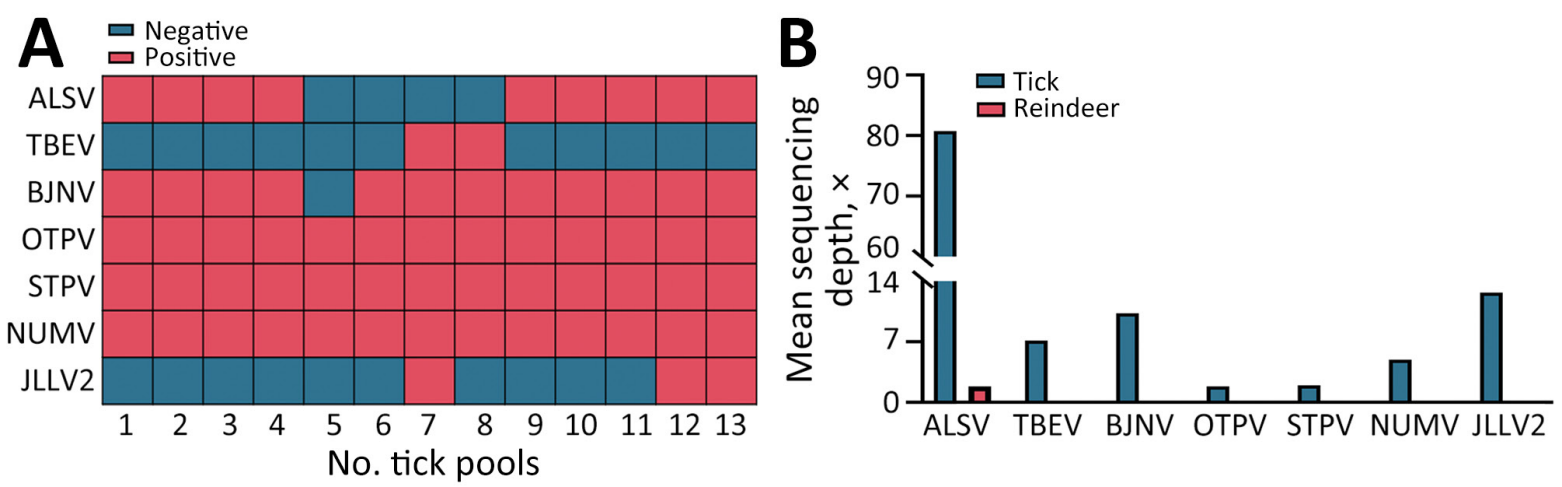
Identified tickborne viruses in study of Alongshan virus infection in *Rangifer tarandus* reindeer, northeastern China. A) Composition of tickborne viruses in tick pools. B) Mean sequencing depth of identified tickborne viruses in libraries. ALSV, Alongshan virus; BJNV, Beiji nairovirus; JLLV2, Jilin luteo-like virus 2; NUMV, Nuomin virus; OTPV, Onega tick phlebovirus; STPV, Sara tick phlebovirus; TBEV, tick-borne encephalitis virus.

Among the tickborne viruses identified in the tick library, ALSV showed the highest mean depth at 80.8×, followed by JLLV2 with a mean depth of 12.7×, BJNV at 10.3×, and TBEV at 7.1×. In contrast, NUMV, OTPV, and STPV displayed the lowest mean depths, measuring 4.9× (NUMV), 1.8× (OTPV), and 1.9× (STPV). Of note, ALSV in the reindeer serum library had a low mean depth of 1.8× (Figure 2, panel B; Appendix Figure 1).

We subsequently confirmed all tickborne viruses identified in this study through seminested reverse transcription PCR (Appendix Table 1). Among the 21 serum samples analyzed, only 1 tested positive for ALSV (4.8%). For the 13 tick pools, each pool exhibited the presence of 3–6 viral species (Figure 2, panel A). Specifically, we consistently detected NUMV, OTPV, and STPV in all tick pools, whereas BJNV was found in 12 pools and ALSV in 9 pools; prevalence rates were 29.5% for BJNV and 16.3% for ALSV. In contrast, JLLV2 and TBEV were only identified in 3 and 2 tick pools, accounting for a prevalence of 3.5% for JLLV2 and 2.2% for TBEV (Appendix Table 2).

Phylogenetic analysis on the basis of the RNA-dependent RNA polymerase gene revealed that all ALSV strains in the Greater Khingan Mountains region clustered together; nucleotide identities ranged from 95.4% to 99.8% (Appendix Figures 2, 3). For the detection of ALSV antibodies, we subjected reindeer serum samples to an indirect ELISA (*2*). Among the 21 samples tested, 7 (33.3%) were positive for ALSV IgG. Of note, 4 of these samples achieved endpoint titers of 320 (Table 2). To further assess neutralizing antibodies against ALSV, we conducted a plaque-reduction neutralization test (*6*) and identified 4 serum samples as ALSV-positive, representing a prevalence of 19.1%; the highest endpoint titer was recorded at 40 (Table 2).

**Table 2:** Serologic detection of Alongshan virus and tickborne encephalitis virus in reindeer, northeastern China*

Sample ID	Reindeer age, y	Reindeer sex	Alongshan virus			Tickborne encephalitis virus	
			ELISA†	PRNT		ELISA†	PRNT
1	>3	M	<20	<20		<20	<20
2	1	M	<20	<20		<20	<20
3	1	M	160	<20		<20	<20
4	2	F	<20	<20		320	80
5	0.5	F	320	20		<20	<20
6	≥3	M	320	20		<20	<20
7	1	M	<20	<20		320	80
8	2	M	<20	<20		320	<20
9	2	F	<20	<20		<20	<20
10	≥3	M	<20	<20		<20	<20
11	≥3	F	20	<20		160	40
12	0.5	F	<20	<20		320	40
13	1	F	<20	<20		320	80
14	2	M	320	20		320	160
15	2	M	80	<20		320	40
16	0.5	M	<20	<20		320	40
17	≥3	M	<20	<20		160	<20
18	2	F	<20	<20		20	<20
19	2	F	<20	<20		80	<20
20	1	M	<20	<20		40	<20
21	1	F	320	40		20	<20

*Antibody titer >20 was considered ELISA- or PRNT-positive. PRNT, plaque-reduction neutralization test. †The ELISA used in our study was specifically targeted against the Alongshan virus VP2 protein.

## Conclusions

The Greater Khingan Mountains, located in northeast China and sharing borders with Russia and Mongolia, boast abundant forest resources, covering as much as 74% of the region (*9*). Over time, the area has gradually transformed into a popular tourist destination during the summer months. Reindeer, serving as a unique and captivating attraction for tourists, increasingly come into close contact with humans. This study detected 3 tickborne viral species (TBEV, ALSV, and BJNV) in ticks that are pathogenic to humans, highlighting the spillover risk for tickborne viruses to humans (*1*,*10*,*11*).

Large wild cervids, such as roe deer, red deer, and reindeer, play a crucial role in the epidemiology of TBEV. In Europe, those cervids are regarded as sentinel species for TBEV because they contribute substantially to tick breeding and activity (*12*). ALSV, an emerging segmented flavivirus, shares several key characteristics with TBEV, including the tick vectors (*I. persulcatus* and *I. ricinus*) and natural foci in China and Europe (*1*–*4*,*6*,*13*).

In this study, ALSV viremia was detected in only 1 reindeer. Conversely, high prevalences of ALSV IgG (33.3%) and neutralizing antibodies (19.1%) were seen (Table 2). This serologic pattern is consistent with observations in animals infected with TBEV, in which seroconversion occurs after a brief viremia after TBEV infection and specific antibodies are rapidly induced and persist for an extended period (*14*). Of note, the study revealed no TBEV viremia in the reindeer, yet a high prevalence of TBEV antibodies (67.7%) was detected (Table 2). Although ALSV and TBEV were not found co-infected in tick pools, neutralizing antibodies to both viruses were detected in 1 reindeer (no. 14) (Table 2; Figure 2, panel A). This finding suggests potential cross-reactivity or exposure to both viruses in this particular reindeer.

Our findings underscore the need to use serologic testing alongside molecular detection in epidemiologic studies concerning ALSV and TBEV in reindeer populations. Moreover, given the substantial reindeer population in the Greater Khingan Mountains and the established practice of monitoring TBEV in reindeer in Europe, this study advocates designating reindeers as wildlife sentinel species for ALSV and TBEV in the Greater Khingan Mountains of northeastern China. Their unique role in tickborne virus epidemiology and close interaction with humans make them invaluable subjects for ongoing surveillance and research efforts in this region.

In conclusion, our study unveiled a diverse array of tickborne viruses in ticks collected from reindeer, substantiating ALSV infection in those animals through both molecular and serologic methods. These findings contribute to our understanding of the tickborne viral ecosystem in northeastern China, highlighting the potential role of reindeer as sentinel animals for the epidemiologic monitoring of ALSV and other emerging tickborne viruses in this region.

Appendix Additional information about Alongshan virus infection in *Rangifer tarandus* reindeer, northeastern China

## References

[1] Wang ZD, Wang B, Wei F, Han SZ, Zhang L, Yang ZT, et al. A new segmented virus associated with human febrile illness in China. N Engl J Med. 2019;380:2116–25. 10.1056/NEJMoa1805068 31141633

[2] Kholodilov IS, Belova OA, Morozkin ES, Litov AG, Ivannikova AY, Makenov MT, et al. Geographical and tick-dependent distribution of flavi-like Alongshan and Yanggou tick viruses in Russia. Viruses. 2021;13:458. 10.3390/v13030458 33799742 PMC7998622

[3] Kuivanen S, Levanov L, Kareinen L, Sironen T, Jääskeläinen AJ, Plyusnin I, et al. Detection of novel tick-borne pathogen, Alongshan virus, in *Ixodes ricinus* ticks, south-eastern Finland, 2019. Euro Surveill. 2019;24:1900394. 10.2807/1560-7917.ES.2019.24.27.1900394 31290392 PMC6628756

[4] Stegmüller S, Fraefel C, Kubacki J. Genome sequence of alongshan virus from *Ixodes ricinus* ticks collected in Switzerland. Microbiol Resour Announc. 2023;12:e0128722. 10.1128/mra.01287-22 36779723 PMC10019301

[5] Ebert CL, Söder L, Kubinski M, Glanz J, Gregersen E, Dümmer K, et al. Detection and characterization of Alongshan virus in ticks and tick saliva from Lower Saxony, Germany with serological evidence for viral transmission to game and domestic animals. Microorganisms. 2023;11:543. 10.3390/microorganisms11030543 36985117 PMC10055853

[6] Wang ZD, Wang W, Wang NN, Qiu K, Zhang X, Tana G, et al. Prevalence of the emerging novel Alongshan virus infection in sheep and cattle in Inner Mongolia, northeastern China. Parasit Vectors. 2019;12:450. 10.1186/s13071-019-3707-1 31511049 PMC6740026

[7] Wang SN, Zhai JC, Liu WS, Xia YL, Han L, Li HP. Origins of Chinese reindeer (*Rangifer tarandus*) based on mitochondrial DNA analyses. PLoS One. 2019;14:e0225037. 10.1371/journal.pone.0225037 31721804 PMC6853604

[8] Liu Z, Li L, Xu W, Yuan Y, Liang X, Zhang L, et al. Extensive diversity of RNA viruses in ticks revealed by metagenomics in northeastern China. PLoS Negl Trop Dis. 2022;16:e0011017. 10.1371/journal.pntd.0011017 36542659 PMC9836300

[9] Meng F, Ding M, Tan Z, Zhao Z, Xu L, Wu J, et al. Virome analysis of tick-borne viruses in Heilongjiang Province, China. Ticks Tick Borne Dis. 2019;10:412–20. 10.1016/j.ttbdis.2018.12.002 30583876

[10] Lu Z, Bröker M, Liang G. Tick-borne encephalitis in mainland China. Vector Borne Zoonotic Dis. 2008;8:713–20. 10.1089/vbz.2008.0028 18837668

[11] Wang YC, Wei Z, Lv X, Han S, Wang Z, Fan C, et al. A new nairo-like virus associated with human febrile illness in China. Emerg Microbes Infect. 2021;10:1200–8. 10.1080/22221751.2021.1936197 34044749 PMC8212832

[12] Michelitsch A, Wernike K, Klaus C, Dobler G, Beer M. Exploring the reservoir hosts of tick-borne encephalitis virus. Viruses. 2019;11:669. 10.3390/v11070669 31336624 PMC6669706

[13] Kholodilov IS, Litov AG, Klimentov AS, Belova OA, Polienko AE, Nikitin NA, et al. Isolation and characterization of Alongshan virus in Russia. Viruses. 2020;12:362. 10.3390/v12040362 32224888 PMC7232203

[14] Klaus C, Ziegler U, Kalthoff D, Hoffmann B, Beer M. Tick-borne encephalitis virus (TBEV) - findings on cross reactivity and longevity of TBEV antibodies in animal sera. BMC Vet Res. 2014;10:78. 10.1186/1746-6148-10-78 24690234 PMC3978054

