# Supplementary material for: Alongshan Virus Infection in Rangifer tarandus Reindeer, Northeastern China
Source: Emerg Infect Dis. 2024 Jul;30(7):1434–7. doi: 10.3201/eid3007.231219 (PMC11210657; doi:10.3201/eid3007.231219)
Supplement: Appendix — Additional information about Alongshan virus infection in Rangifer tarandus reindeer, northeastern China [file 23-1219-Techapp-s1.pdf]

Article DOI: https://doi.org/10.3201/eid3007.231219

EID cannot ensure accessibility for supplementary materials supplied by authors. Readers who have difficulty accessing supplementary content should contact the authors for assistance.

Alongshan Virus Infection in *Rangifer tarandus* Reindeer, Northeastern China

Appendix

Appendix Table 1. Primers used in the present study

Virus	Primer	Position (bp)	Sequence (5′→3′)	Polarity	Amplicon (bp)
Alongshan virus					
	ALSV F	345	CCCTCGAAAGGYTATGACA	+	333
	ALSV R1	773	CTTCCCAGTCTTGACCTGC	-	
	ALSV R2	678	GTTGCATCATCCAYGCATC	-	
Tick-borne encephalitis virus					
	TBEV F	152	TGTGGCTTGACTCCATCTACCAG	+	289
	TBEV R1	564	GGCACAACAAGGACACGTCT	-	
	TBEV R2	441	TGCGGCTCTACTTTAACCGTG	-	
Beiji nairovirus					
	BJNV F	3333	TAACCACCTGCATTTTAGTCC	+	417
	BJNV R1	3777	TCCATTAGGGTAAAGTGCAT	-	
	BJNV R2	3750	TCTCAAGGCACTTATCAACCA	-	
Nuomin virus					
	NUMV F	748	AGGCTATCATATTCATGTGGT	+	303
	NUMV R1	1099	GTCCTCGATTGCATAACGAAC	-	
	NUMV R2	1051	GTTCCGCTCCTCATTAACCTT	-	
Onega tick phlebovirus					
	OTPV F	1792	TCCTGAAGAAGCTACGGCAT	+	343
	OTPV R1	2241	GCAGTTTCCCCAACACCTT	-	
	OTPV R2	2135	TTGTCTTCCAGCAGGACCATG	-	
Sara tick phlebovirus					
	STPV F	1153	CCTTCAGGCATTGGTCCAGT	+	356
	STPV R1	1620	AACTCCGTCCTTGTGTAGAGC	-	

Virus	Primer	Position (bp)	Sequence (5′→3′)	Polarity	Amplicon (bp)
	STPV R2	1509	CAAGTAAGCCCTCAATGTCTCC	-	
Jilin luteo-like virus 2					
	JLLV2 F	1491	CTTGGCATTTCCGACAACGTTT	+	425
	JLLV2 R1	1960	CAGTCCCAGCACGACTTGTCA	-	
	JLLV2 R2	1916	ACGAGCTCTCACAAGTCGGTA	-	

Appendix Table 2. Detection of tick-borne viruses in tick pools collected from reindeers.

Virus	Sequence length†	Proportion (%) ‡	Female tick pools (No. positive/Total)	Male tick pools (No. positive/Total)	Prevalence (%) *	Confidence intervals (95%)
ALSV	Segment 1: 3,014 bp	Segment 1: 98.3	4/8	5/5	16.3	8.0–31.7
	Segment 2: 2,718 bp	Segment 2: 96.6				
	Segment 3: 2,766 bp	Segment 3: 98.5				
	Segment 4: 2,716 bp	Segment 4: 99.8				
TBEV	10, 756 bp	96.9	2/8	0/5	2.2	0.4–6.9
BJNV	L segment: 13,665 bp	L segment: 92.1	7/8	5/5	29.5	16.4–62.2
	S segment: 2,753 bp	S segment: 74.2				
OTV	L segment: 271 bp	L segment: 4.1	8/8	5/5	100.0	–
	S segment: 255 bp	S segment: 13.3				
STV	L segment: 1, 005 bp	L segment: 15.0	8/8	5/5	100.0	–
NUMV	9, 472 bp	86.9	8/8	5/5	100.0	–
JLLV2	2, 318 bp	86.9	1/8	2/5	3.5	1.0–9.6

† The length of sequence that obtained from metatranscriptomic analysis.

‡ The proportion of genome length obtained by metatranscriptomic analysis to the total genome sequence length of the viruses.

* Prevalence of tick-borne virus infection in ticks was calculated by using PooledInfRate Excel Add-In version 4.0 (Microsoft, https://www.microsoft.com) and a 1-sample analysis with a bias-corrected maximum likelihood estimation method with 95% Confidence intervals and a scale of 100.

Appendix Table 3. The reference viruses of the Jingmenvirus group used in the present study

Virus	Strain	GenBank accession number
Alongshan virus	NE-TH4	ON408067
Alongshan virus	H3	MH158415
Alongshan virus	HLJ1	MT514916
Alongshan virus	HLJ2	MT514917
Alongshan virus	Kuutsalo-23	MN107156
Alongshan virus	Haapasaari-18	MN107160
Alongshan virus	Miass527	MN648770
Alongshan virus	Miass502	MW525314
Alongshan virus	Miass519	MN648774
Alongshan virus	Miass506	MW525318
Takachi virus	IM-OI70	LC628194
Jingmen Tick Virus	SY84	NC024114
Yanggou tick virus	XJ-YGTV-1	MT248420
Xinjiang tick virus 1	XJO381	MZ244282
Heilongjiang tick virus	HLJ41	MK721862
Guangxi tick virus	GX46	MK721858
Mogiana tick virus	Yunnan2016	MT080097

Appendix Table 4. Nucleotide sequences of the identified viruses in the present study

Library	Viral species	Gene*	Viral strain	GenBank accession No.
Tick				
	Alongshan virus	RdRp	GH1	OQ185286
	Alongshan virus	RdRp	GH2	OQ185287
	Alongshan virus	RdRp	GH3	OQ185288
	Alongshan virus	RdRp	GH4	OQ185289
	Tick-borne encephalitis virus	E protein	GH1	OQ185284
	Tick-borne encephalitis virus	E protein	GH2	OQ185285
	Beiji nairovirus	RdRp	GH1	OQ185280
	Sara tick phlebovirus	RdRp	GH1	OQ185283
	Onega tick phlebovirus	RdRp	GH1	OQ185282
	Nuomin virus	RdRp	GH1	OQ185281
	Jilin luteo-like virus 2	RdRp	GH1	OQ185279
Reindeer				
	Alongshan virus	RdRp	RD1	OQ743416

*RdRp, RNA-dependent RNA polymerase; E protein, envelope protein.

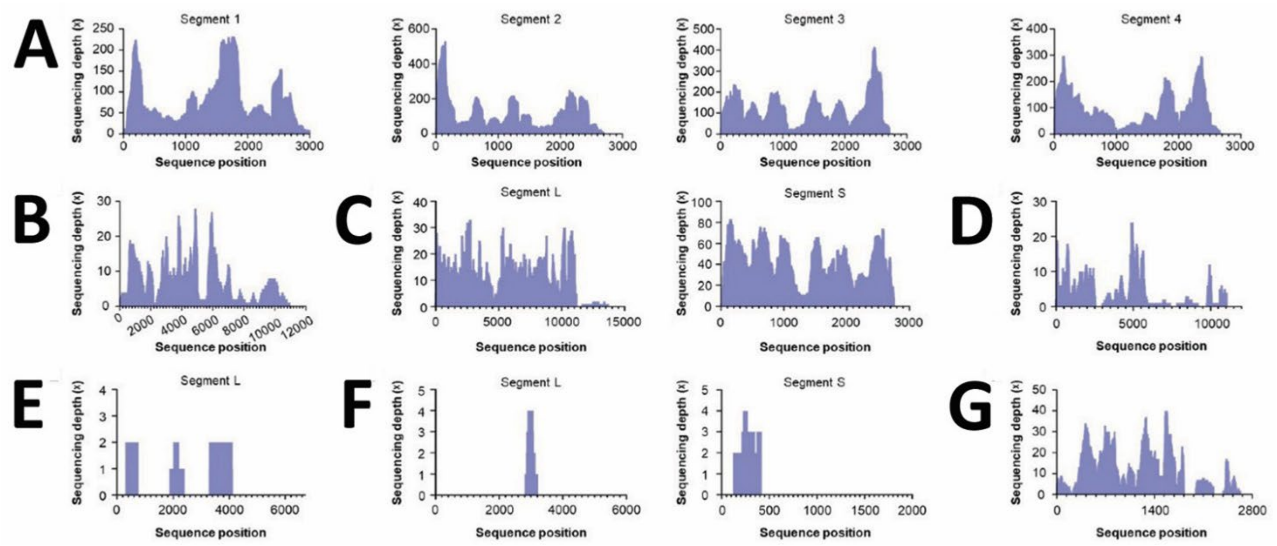

Appendix Figure 1. The mapped read count plots of the sequenced tick-borne viruses' genomes. (A) Alongshang virus; (B) Tick-borne encephalitis virus; (C) Beiji nairovirus; (D) Sara tick phlebovirus; (E) Onega tick phlebovirus; (F) Nuomin virus; (G) Jilin luteo-like virus 2. The histograms illustrate the depth of coverage for each base of the viral genome.

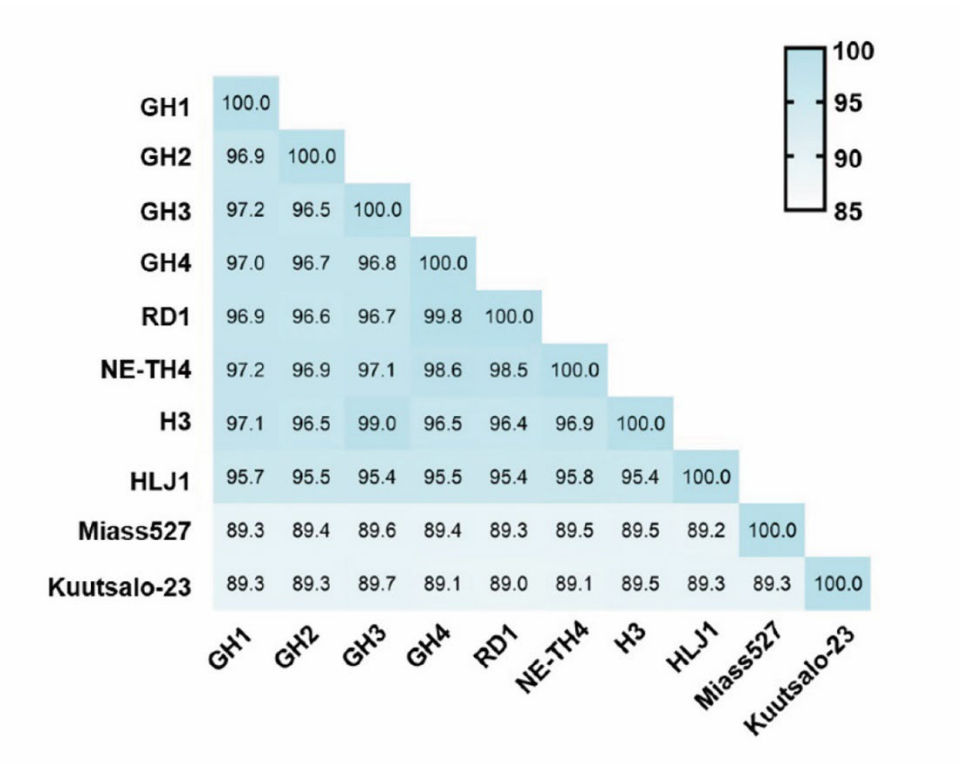

Appendix Figure 2. Sequence identity of the identified Alongshan virus by comparing with the reference strains. The analysis was based on the nucleotide sequences of the RNA-dependent RNA polymerase gene of the representative strains of Alongshan virus. Details of all reference sequences are available in Appendix Table 3.

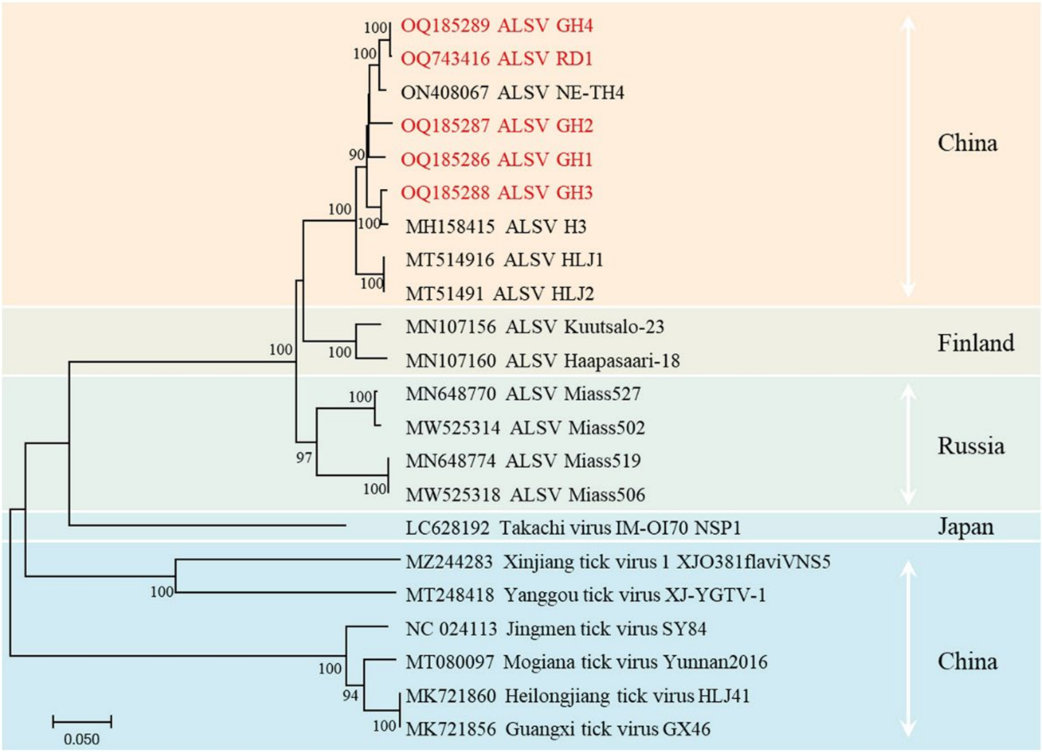

Appendix Figure 3. Phylogenetic analysis of the identified Alongshan virus. The analysis based on the nucleotide sequences of the RNA-dependent RNA polymerase gene of Jingmenviruses was conducted using the maximum likelihood method within MEGA 7.0 software. A bootstrapping analysis of 1,000 replicates were performed, and values greater than 70 were considered significant and displayed in the tree. All virus strains obtained in this study are highlighted in red to distinguish them from the reference sequences. The details of all reference sequences are available in Appendix Table 3.
